# Characterization of Iron Core–Gold Shell Nanoparticles for Anti-Cancer Treatments: Chemical and Structural Transformations During Storage and Use

**DOI:** 10.3390/ma11122572

**Published:** 2018-12-17

**Authors:** Ya-Na Wu, Dar-Bin Shieh, Li-Xing Yang, Hwo-Shuenn Sheu, Rongkun Zheng, Pall Thordarson, Dong-Hwang Chen, Filip Braet

**Affiliations:** 1 Institute of Oral Medicine and Department of Stomatology, College of Medicine, National Cheng Kung University Hospital, National Cheng Kung University, Tainan 70101, Taiwan; dbshieh@mail.ncku.edu.tw; 2 Institute of Biological Chemistry, Academia Sinica, Taipei 11529, Taiwan; 3 Center for Micro/Nano Science and Technology, Advanced Optoelectronic Technology Center, Innovation Center for Advanced Medical Device Technology, National Cheng Kung University, Tainan 70101, Taiwan; 4 Institute of Basic Medical Sciences, National Cheng Kung University, Tainan 70101, Taiwan; fingerzoo@gmail.com; 5 National Synchrotron Radiation Research Center, Hsinchu Science-Based Industrial Park, Hsinchu 30076, Taiwan; hsheu@nsrrc.org.tw; 6 Australian Centre for Microscopy & Microanalysis, The University of Sydney, Sydney, NSW 2006, Australia; rongkun.zheng@sydney.edu.au; 7 School of Chemistry, The University of New South Wales, Sydney, NSW 2052, Australia; p.thordarson@unsw.edu.au; 8 Department of Chemical Engineering, National Cheng Kung University, Tainan 70101, Taiwan; chendh@mail.ncku.edu.tw; 9 School of Medical Sciences—The Bosch Institute, The University of Sydney, NSW 2006, Australia; filip.braet@sydney.edu.au

**Keywords:** biocompatibility, cancer, degradation, nanoparticles, zero-valent iron

## Abstract

Finding a cancer-selective drug that avoids damaging healthy cells and organs is a holy grail in medical research. In our previous studies, gold-coated iron (Fe@Au) nanoparticles showed cancer selective anti-cancer properties in vitro and in vivo but were found to gradually lose that activity with storage or “ageing”. To determine the reasons for this diminished anti-cancer activity, we examined Fe@Au nanoparticles at different preparation and storage stages by means of transmission electron microscopy combined with and energy-dispersive X-ray spectroscopy, along with X-ray diffraction analysis and cell viability tests. We found that dried and reconstituted Fe@Au nanoparticles, or Fe@Au nanoparticles within cells, decompose into irregular fragments of γ-F_2_O_3_ and agglomerated gold clumps. These changes cause the loss of the particles’ anti-cancer effects. However, we identified that the anti-cancer properties of Fe@Au nanoparticles can be well preserved under argon or, better still, liquid nitrogen storage for six months and at least one year, respectively.

## 1. Introduction

One of the pressing healthcare needs worldwide is the development of cancer treatments that have greater efficacy and reduced toxicity [1,2,3,4]. It is of genuine clinical value to develop biocompatible anti-cancer drugs that selectively target cancer cells but have lower toxicity to healthy cells and organs. One promising anti-cancer material is nanoscale iron. This type and other magnetic nanoparticles have attracted significant attention in the discipline of nanomedicine because their ideal size and magnetic characteristics suggest considerable potential as novel theragnostic (i.e., combined therapeutic-and-diagnostic) agents. Thus far, among the magnetic nanomaterials, iron oxides have been found to be biocompatible and are being extensively applied in nanomedicine research as well as clinical application [5,6]. Elemental iron, sometimes called zero-valent iron or Fe(0), has better magnetic susceptibility than iron oxides, which suggests excellent potential for biomedical applications. For example, it has been reported that Fe(0) nanoparticles are bactericidal in culture condition at concentrations as low as a few milligrams per liter [7,8] and such nanoparticles have also demonstrated remarkable effectiveness in the breakdown of organic contaminants to near-zero concentrations in environmental remediation [9,10,11]. To date, however, less attention has been given to the direct interaction between Fe(0) and living tissue cells or the human body. In the limited research reports, researchers focused on the direct interaction of Fe(0) nanoparticles and blood cells [12,13] as well as bronchial cells [14].

**Scheme 1:**
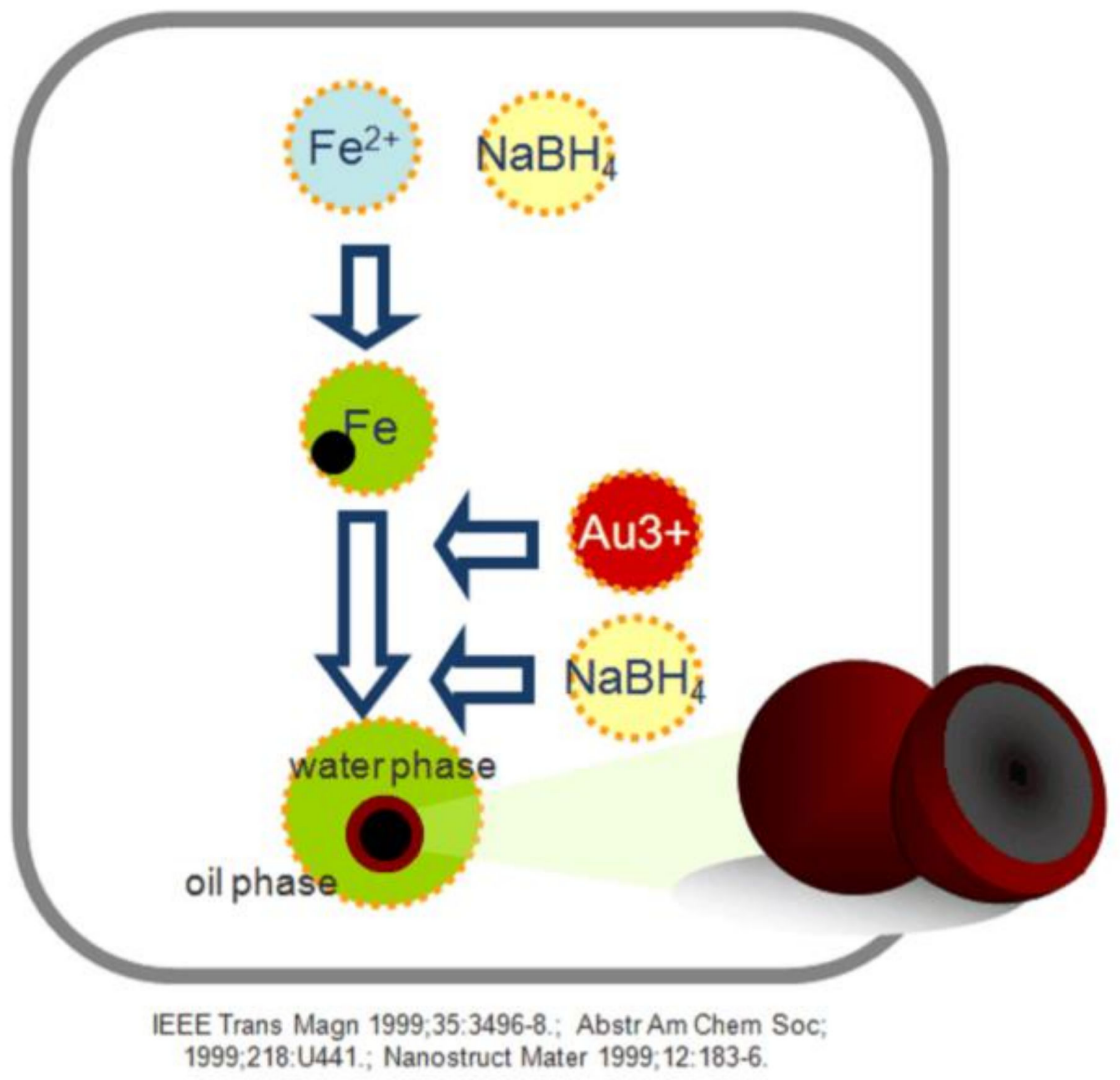
Scheme depicting the different steps involved in the synthesis of Fe@Au nanoparticles as employed in our studies.

**Scheme 2:**
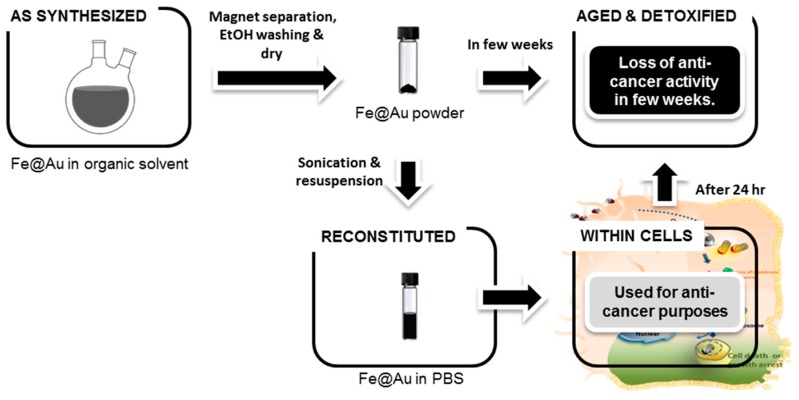
Schematic illustration of Fe@Au synthesis, reconstitution and the ageing or “detoxifying” process. Fe@Au is synthesized in a water-in-oil reverse micelle system, which is nominated as “as synthesized” Fe@Au. Next, as-synthesized Fe@Au is magnetically separated, washed in ethanol (EtOH) and dried. Prior to use, the nanoparticles are reconstituted in phosphate-buffered saline for anti-cancer application, which is described as “reconstituted” Fe@Au. The cancer cells are treated with reconstituted Fe@Au and the internalized Fe@Au is denoted as “within cells”. Notably, incorporation of Fe@Au in cells for more than 48 h or Fe@Au powder stored for more than 2 weeks lose its anti-cancer effect and we defined such Fe@Au without anti-cancer activity as “aged” or “detoxified” Fe@Au.

Despite the extensive potential biomedical applications, iron nanoparticles suffer from oxidation and instability. The iron core-gold shell nanoparticles, denoted as Fe@Au, were initially designed two decades ago by Zhou et al. [15]. Briefly, Fe@Au are prepared inside the reverse micelles through the reduction of metal salts using sodium borohydride (Scheme 1). Such core-shell structure is proposed to take advantage of magnetic susceptibility of zero valent iron cores and the passivating properties of the gold coatings, which delays the oxidation of the iron cores [16]. In addition, Fe@Au nanoparticles has also been proposed as imaging probes for magnetic resonance imaging [17], X-ray computed tomography [17,18] and so forth. Previously, we reported that freshly prepared nanoparticles with an iron core and gold shell (denoted “Fe@Au”) have potent anti-cancer properties, selectively suppressing the growth of various cancer cells, while sparing healthy cells. We have demonstrated this effect in 12 normal and cancer cell lines, including primary cultured cells and within relevant in vivo (animal model) settings [19,20]. Typically, the Fe@Au nanoparticles have an average diameter of 10 nm with a shell of face-centered-cubic Au and body-centered cubic Fe(0), without traces of iron oxide [19,21]. We have demonstrated that Fe@Au triggers mitochondria-mediated autophagy and halts cancer cell growth, in oral cancer cells [21] and provokes an alternative cytotoxic pathway in colorectal cancer cells [20]. Given our evidence that the cytotoxicity of Fe@Au is linked to the redox activity of the Fe(0), it is not surprising that cytotoxicity decreases with the progression of oxidation of iron in the particles, a process we termed “ageing” [19]. This phenomenon also occurs when storing freshly prepared Fe@Au particles over time. Clearly then the structure–cytotoxicity relationship of Fe@Au at different stages (Scheme 2) and the optimal storage condition are vital issues for practical therapeutic application in the near future. 

Considering all of the above challenges, we explored in this study: (1) the structural differences of freshly synthesized Fe@Au, reconstituted Fe@Au and Fe@Au incubated together with cells; (2) the structural and compositional change and the biocompatibility of aged Fe@Au with time; and (3) the optimal storage conditions of Fe@Au nanoparticles to preserve their selective anti-cancer activity.

## 2. Materials and Methods 

### 2.1. Materials

Phosphate-buffered saline (PBS), RPMI-1640, Keratinocyte-SFM medium, fetal bovine serum (FBS), L-glutamine and Antibiotic-Antimycotic (100×) liquid were obtained from Invitrogen (Carlsbad, CA, USA). Premixed WST-1 Cell Proliferation Reagent was purchased from Clontech (Mountain View, CA, USA). All other chemicals used in this study were of analytical grade and obtained from Sigma-Aldrich (St. Louis, MO, USA).

### 2.2. Synthesis of Fe@Au Nanoparticles

The core–shell structures were synthesized by using a sequential synthesis technique within reverse micelles in a cetyltrimethylammonium bromide/N-butanol/water system [15]. Briefly, all reverse micelle solutions were prepared with 1.9 M cetyltrimethylammonium bromide as the surfactant and isooctane as the oil phase, under argon gas. In the first step, two micellar solutions were combined: 0.2 M FeSO_4_ (aq) and 0.5 M NaBH_4_ (aq), both solutions with a molar ratio (ω) of water to surfactant of 2.54, using a magnetic stirrer at a speed of 260 rpm for 30 min to form the iron cores. In the second step, a coating of gold was applied onto the iron core by adding 0.05 M HAuCl_4_ and 0.8 M NaBH_4_ reverse micellar solutions (ω = 3.8) after completion of the first reaction. 

To purify Fe@Au from the synthetic process above, the nanoparticles were ultrasonically washed in ethanol and subsequently magnetically collected and this process was repeated three times. Particles were then prepared for storage under one of three conditions: vacuum-dried (30 min at 25 °C) particles stored in air; vacuum-dried particles stored in argon-filled vials at room temperature; the washed particles redispersed (with sonication) immediately into a suspension of 100% ethanol, at a concentration of 10 mg/mL and then frozen in liquid nitrogen. To reconstitute any of the dried Fe@Au nanoparticles for experiments, the particle powder was resuspended in water at a final concentration of 10 mg/mL and sonicated for 3 min before use. The nanoparticle suspension stored in liquid nitrogen was thawed and then diluted in PBS to assigned concentrations.

### 2.3. Characterization of Fe@Au

Dried Fe@Au nanoparticles were dispersed in absolute ethanol by sonication and then dropped onto copper grids with carbon-film support (GSCu300FL, Proscitech, Queensland, Australian) and dried. The specimens were then observed with a TEM (3000F, JEOL, Tokyo, Japan) operating at 300 keV accelerating voltage, equipped with a SC1000 11-megapixel CCD camera (Gatan, CA, USA) and an energy-dispersive X-ray spectroscopy (EDXS) system (INCAEnergy, Oxford Instruments, Oxfordshire, UK).

### 2.4. X-ray Diffraction Analysis of Fe@Au

X-ray diffraction (XRD) patterns of Fe@Au powder samples were obtained on a BL01C2 beam-line at the National Synchrotron Radiation Research Center (NSRRC), Taiwan, with a wavelength of 0.77495 Å (16 keV). The synchrotron beam (0.15 mm size) was produced by a 5.0 T superconducting wavelength shift magnet and was wavelength selected with a double-crystal monochromator based on the Si (111) plane and focused with a refocusing toroidal mirror. Powder diffraction patterns were recorded using a fixed Mar345 imaging plate detector at a distance of 260 mm from the sample under ambient temperature. The X-ray exposure time is circa 5 min.

### 2.5. Cell Culture

The cancer cell-line OECM1, which we used for testing the efficacy of the nanoparticles, was established from a Taiwanese male oral cancer patient with a history of chewing areca quid. OECM-1 was kindly provided by Dr Kuo-Wei Chang (Institute of Oral Biology, National Yang-Ming University, Taiwan). The cancer cell-line OECM1 was grown in RPMI-1640 supplemented with 10% FBS [22], in a humidified incubator at 37 °C with 5% CO_2_. We also isolated and cultured human normal oral keratinocytes (hNOK), to use as control cells, according to the method of Kim et al. [23], following a protocol approved by the University of Sydney (No. #08E011) and University of Cheng-Kung Internal Review Board for Ethics & Human Research (No. #97218). Transformed non-malignant cells VERO and NIH/3T3 were obtained from the American Type Culture Collection (ATCC, Manassas, Virginia, USA) and were kept in Dulbecco’s Modified Eagle’s Medium (DMEM) with 10% fetal bovine serum. The cells were cultured and maintained according to the guidelines of the ATCC.

### 2.6. WST-1 Cell Viability Assay

To evaluate the cytotoxicity of Fe@Au nanoparticles, cells in their log-phase were seeded at a density of 5000 cells per well in a 96-well culture plate. Prior to each in vitro experiment, the assigned particles were freshly prepared in PBS, from dried stock or after recovery from defrosted ethanol suspension that had been stored in liquid nitrogen, at a concentration of 10 mg/mL. Cells were then cultured with the assigned concentration of nanoparticles from the relevant stock suspension for 48 h, after which the cells were tested for viability with a WST-1 assay. The optical absorbance at 450 nm was recorded with a microplate reader (Sunrise Absorbance Reader; Tecan, Männedorf, Switzerland) after the cells had been incubated with WST-1 reagent for 1 h. The 50% inhibition concentration (IC_50_) was then calculated by linear interpolation or linear extrapolation according to the cell viability results.

### 2.7. Transmission Electron Microscopy of Treated Cells 

Fe@Au-exposed cells were grown on 13 mm-diameter plastic coverslips and then fixed in 2.5% glutaraldehyde in PBS for 1 h at room temperature. After washing with fresh PBS, the cells were incubated in 1% osmium tetroxide in PBS for 1 h at room temperature. Samples were washed in fresh PBS, followed by gradual dehydration in a graded ethanol series of 50%, 70%, 90%, 95% and 100% (1 h per stage). The dehydrated samples were then infiltrated in stages with resin–ethanol solutions containing 50%, then 75% and then 100% resin (4 h per stage). Next, samples were polymerized at 50 °C for 24 h. The embedded cells were cut using an ultramicrotome (Ultracut S, Leica Microsystems, Northern Sydney, NSW, Australia) into 80 nm ultrathin sections by using a diamond knife and then post-stained with uranyl acetate and lead citrate. Transmission electron microscopy was carried out on a JEOL JEM-1400 at 80 keV or the JEOL 3000 F (at 300 keV) used for characterization of the nanoparticles. 

### 2.8. Statistical Analysis

All represented data are expressed as mean ± standard error. Statistical differences were evaluated by using the Student *t*-test or ANOVA. Results were considered statistically significant at the 95% confidence interval (i.e., *p* < 0.05) but we have provided all the *p*-values. All figures shown in this article were obtained from at least three independent experiments (i.e., full replicates).

## 3. Results

### 3.1. Structural and Chemical Transformations of Fe@Au In Vitro and In Vivo

**Figure 1:**
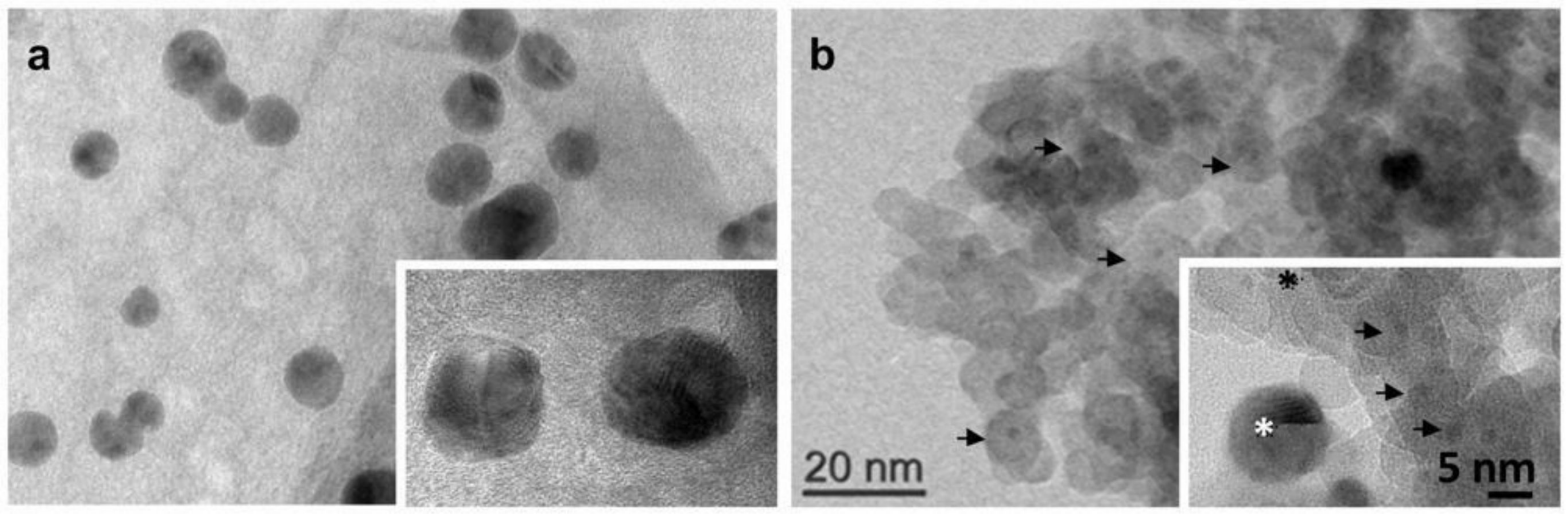
TEM images of freshly synthesized and reconstituted Fe@Au nanoparticles. Compared to the as-synthesized nanoparticles (**a**), the reconstituted nanoparticles (**b**) lose their homogeneity and show a more uneven electron density. The composition was analyzed by EDXS as shown in Table 1. The dark clump, marked by the white star, is Au and the major composition of low-electron-density aggregates, marked by black star, is Fe and O (i.e., iron oxide; see Supplementary Materials, Table S1). The black arrows indicate pale ring-like structures. The magnification and the scale of figure (**a**) is identical to figure (**b**).

To confirm the viability of the freshly made nanoparticles before examining their changes during storage and in use, we first examined their structures (see Figure 1a) and tested their biological activity. To do so, the oral cancer cells line OECM1 and normal oral keratinocytes (hNOK), which were paired healthy primary cells, were used to evaluate the selective anti-cancer property of a newly made batch of Fe@Au. The cytotoxic profiles of Fe@Au nanoparticles clearly differ markedly between OECM1 and hNOK (Figure S1, *p* = 0.0053, Student *t*-test analysis between the largest dose difference; that is at dose of 50 µg/mL): about 80% of the cancer cells were killed at low doses (~10 µg/mL) of the nanoparticles, while the normal cells were unaffected until much higher doses (~100 µg/mL). These cytotoxic effects accorded with our previous observations [19,20] in a series of oral and colon rectal cancer cell lines and paired healthy cells in vitro and in vivo. Hence, this experimental Fe@Au condition was used for the next stage of the study, which considered the effects of washing, drying and redispersing on the structure and cytotoxic efficacy of the nanoparticles.

For any future clinical applications of Fe@Au, any residual surfactants and organic solvents from the nanoparticle synthesis should be removed by, for example, magnetic separation, washing and vacuum drying of the particles, to avoid non-specific cytotoxicity that can be caused by residual surfactants and solvent [24]. In our previous work, we stored the washed and vacuum-dried nanoparticles in oxygen-poor conditions and then, before use, the nanoparticles were resuspended in PBS by sonication; these redispersed nanoparticles we termed “reconstituted Fe@Au”. Given that surfactant removal and drying can adversely affect nanoparticle structure and cause agglomeration [25], we always observed freshly synthesized and reconstituted nanoparticles by TEM before use for quality-assurance purposes. Figure 1a is a typical TEM image of as-synthesized particles and it is evident that the particles are approximately spherical in morphology and have a relatively narrow size distribution. Energy-dispersive X-ray spectroscopy (EDXS) confirmed the homogenous composition of the particles, with measured particles giving an average composition of 47.9% Fe and 52.1% Au (Table 1a). This approximates the composition of 43% Fe and 57% Au that would be expected based on the atomic absorption analysis previously reported [19]. After the surfactant removal, Fe@Au were stored under argon at room temperature for reconstitution within two weeks. However, the washing and/or storage process affects the particles, as is clear in Figure 1B where aggregation of reconstituted particles is evident, along with changes in morphology and more-uneven electron dense contrast. To confirm that the changes in contrast represented compositional variation (i.e., mass contrast), rather than diffraction contrast, we used EDXS to characterize the electron-dense particle in Figure 1B (white asterisk), which revealed that they are enriched in Au, containing approximately 88%Au and 12% Fe (Table 1b). Examining a less-dense area in Figure 1b (black asterisk), we found that Fe was the major element, while it only contained about 1.7% of Au (Table 1c). Intriguingly, ring-like pale layers around the dense core (black arrows) were observed. To further verify whether the elemental iron of Fe@Au is fully oxidized after reconstitution, EDXS was next used to evaluate the relative proportions of Au, Fe and O in reconstituted samples and in aged Fe@Au. These results (Supplementary Materials, Table S1) show that reconstituted Fe@Au contain less O (*p* = 0.0001), though still a large proportion. These data suggest that the Fe in reconstituted samples are less oxidized than aged Fe@Au and therefore potentially retains a proportion of Fe(0) that provide the cytotoxic effects in cancer cells.

**Figure 2:**
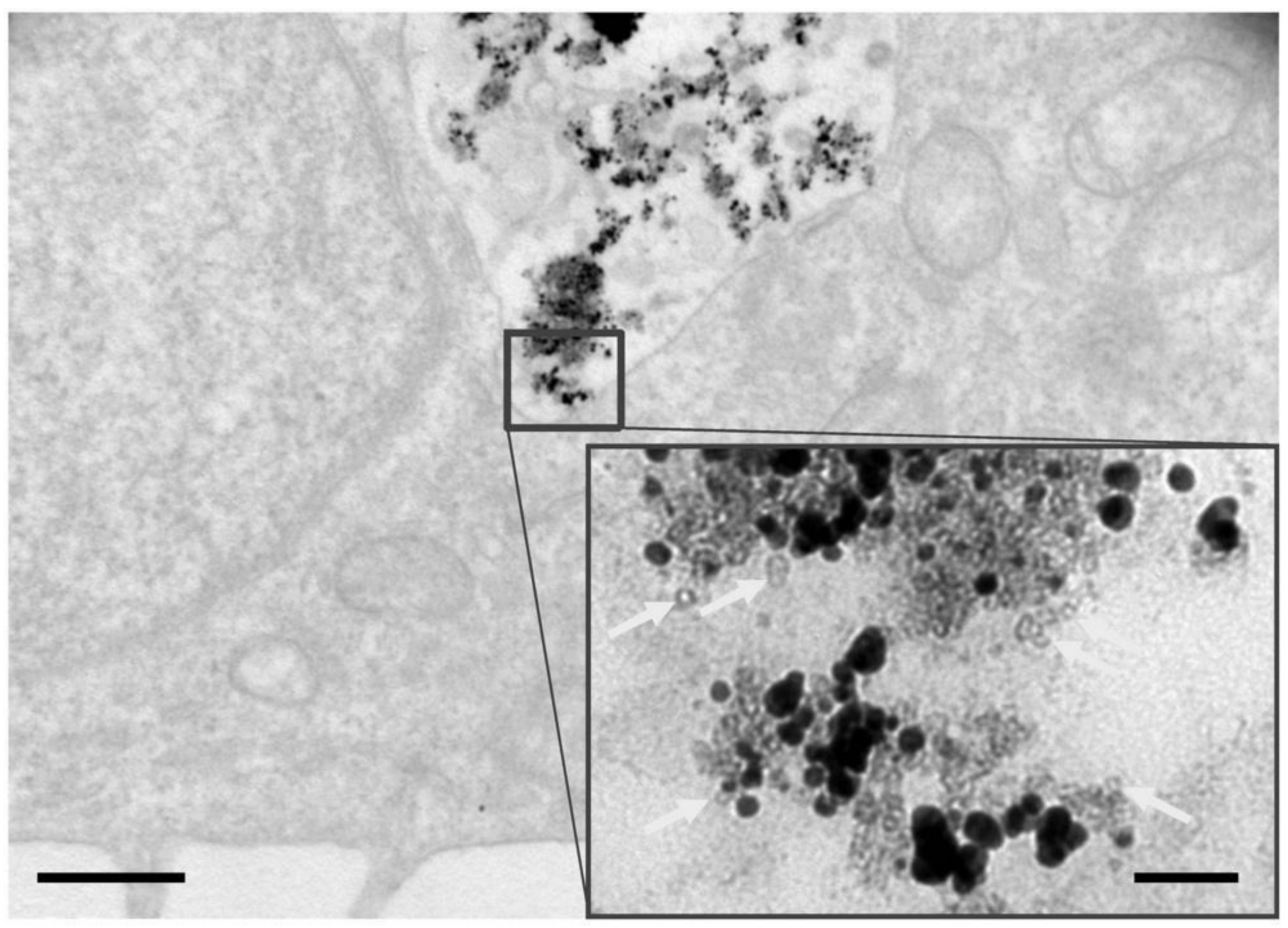
TEM images of Fe@Au within cells, internalized by human oral cancer cells. The structure of reconstituted Fe@Au internalized by human oral cancer cells is disrupted and loses the core–shell structure of as-synthesized Fe@Au. The morphology of internalized Fe@Au is similar to the reconstituted Fe@Au but the pale, ring-like structures of Figure 1b are not apparent. Instead, donuts-like rings are evident and marked by white arrows. The left scale bar is 500 nm and the right scale bar of the inserted figure is 50 nm.

For comparison, Figure 2 is a micrograph of freshly prepared Fe@Au nanoparticles 24 h after being internalized by cancer cells (OECM1). There are larger particles of dark contrast, which would seem likely to be agglomerated Au, consistent with the structures seen in Figure 1b and many lighter particles that appear to contain central void structures. As we discuss later, these are likely to be the iron-oxide shells that form from the original Fe cores of the Fe@Au nanoparticles during oxidation within the cancer cells.

### 3.2. Aging of Fe@Au Nanoparticles and Their Diminished Anti-Cancer Activity

**Figure 3:**
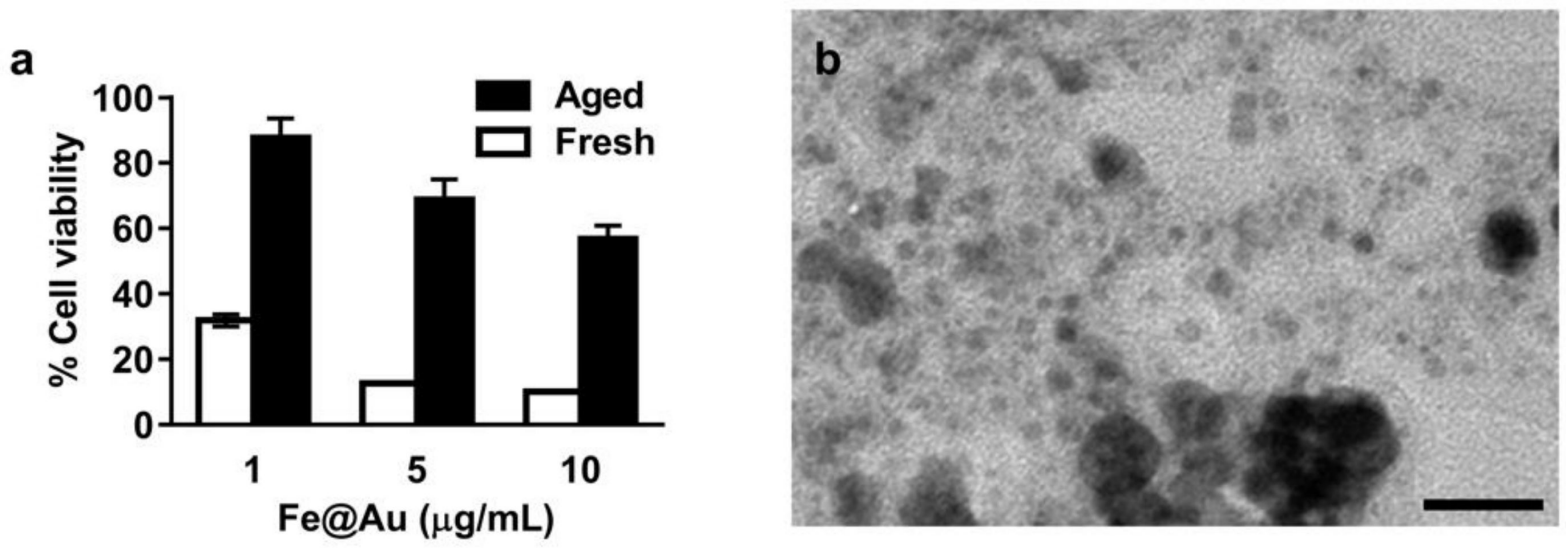
The aged Fe@Au forms irregular debris and large clumps under TEM. (**a**) The cell viability assay used OECM1 oral cancer cells that were exposed to aged or freshly reconstituted Fe@Au for 48 h and examined by the WST-1 assay. As is evident, the IC_50_ of aged Fe@Au is higher than 10 μg/mL and therefore we classified the Fe@Au as “aged”; (**b**) The electron micrograph of aged Fe@Au shows that the particles are decomposed into large clumps and pale debris. Some small donut-shaped debris is also observed.

Our previous study briefly reported that the IC_50_ of Fe@Au keeps increasing with storage time after synthesis, corresponding to a decrease in cytotoxicity with storage [19]. Once the IC_50_ of Fe@Au in oral cancer cells, OECM1, rose higher than 10 µg/mL, we considered the Fe@Au batch was aged (denoted as “aged Fe@Au”) and we no longer used those nanoparticles for further experiments (Figure 3a, *p* < 0.0001, ANOVA). Understanding and therefore being able to minimize, this ageing process is essential if these nanoparticles are to be used for future medical applications. 

**Figure 4:**
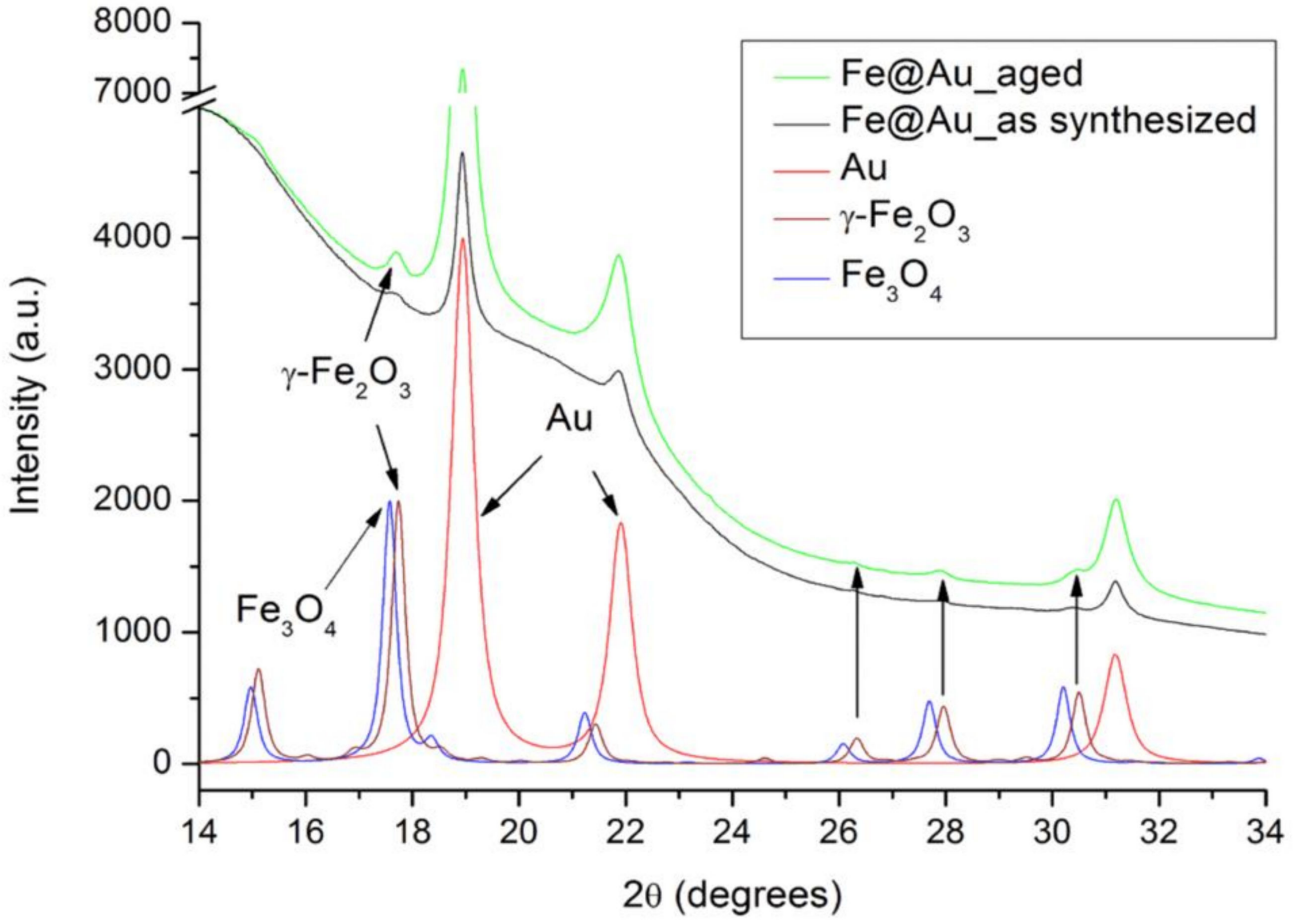
The compositional change of reconstituted and aged Fe@Au analyzed by synchrotron radiation XRD. Both the reconstituted and aged Fe@Au showed iron oxide peaks corresponding to γ-Fe_2_O_3_ diffraction peaks, not Fe_3_O_4_. (Note that the traces for Au, γ-Fe_2_O_3_and Fe_3_O_4_ are all XRD standard curves from The Powder Diffraction File.) The reconstituted Fe@Au shows one small peak that matches γ-Fe_2_O_3_ signals, whereas the aged Fe@Au shows intensified γ-Fe_2_O_3_ signals. Calculations according to the Scherrer equation indicate that the average size of γ-Fe_2_O_3_in reconstituted Fe@Au is 5.02 nm and the diameter of γ-Fe_2_O_3_ in the aged Fe@Au is approximately 11.20 nm.

To that end, we characterized aged Fe@Au nanoparticles to investigate the mechanism behind the aging and the corresponding decrease in cytotoxicity. Differences are evident from the TEM images of aged Fe@Au (Figure 3b) when compared with freshly synthesized nanoparticles (Figure 1a); in particular the aged Fe@Au sample contains large clumps along with small debris of low electron-density. We analyzed the phases and average particle (crystallite) sizes of the fresh and the aged Fe@Au by XRD (Figure 4). The major XRD peaks of fresh and aged Fe@Au correspond to the diffraction peaks for elemental Fe and Au. Both the reconstituted and the aged Fe@Au show minor peaks of γ-Fe_2_O_3_ (rather than Fe_3_O_4_), although clearly the area of the γ-Fe_2_O_3_ diffraction peaks is larger in the aged nanoparticles, consistent with increased oxidation of the iron-rich cores. Given that the major diffraction peaks for elemental Fe and Au tend to overlap [26], we synthesized and scanned pure Fe nanoparticles for comparison; the diffraction pattern for these Fe nanoparticles is identical with the standard diffraction file of bulk Fe (see Supplementary Materials, Figure S2). The XRD diffraction peak at 2θ = 17.698 is corresponded to (111) crystal face reflection of γ-Fe_2_O_3_ (see Figure 4)_._ The full width at half maximum (FWHM) are 0.805 and 0.361 degree in 2θ by peak profile fitting in Lorentzian function for freshly reconstituted and the aged Fe@Au respectively. The calculation of particle size was based on the Scherrer’s equation [27,28,29],

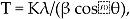
(1)
where K is the shape factor, λ is the x-ray wavelength, β is the full width at half maximum intensity in radians, θ is the Bragg angle and τ is the mean size of the nanoparticles [28,29]. The Scherrer equation is limited to nanoscale particles and the dimensionless shape factor has a typical value of about 0.9 for spherical nanoparticles. The wavelength of X-rays from the XRD experiment is λ = 0.77496 Å. Therefore the calculated particle size are 5.02 nm and 11.20 nm for freshly reconstituted and the aged Fe@Au respectively. To confirm that these measurements represent particle sizes (rather than just crystallite sizes in larger particles), we undertook direct measurements of whole nanoparticles from our electron micrographs. The mean size of freshly reconstituted particles was found to be 5 ± 1.1 nm (*n* = 67) while the mean size of aged Fe@Au was determined as 8 ± 3.4 nm (*n* = 69). Thus, the expansion of Fe@Au upon ageing was obvious from both XRD analysis and direct measurement under TEM and this increased size is consistent with oxidation of the nanoparticles, as discussed below.

**Figure 5:**
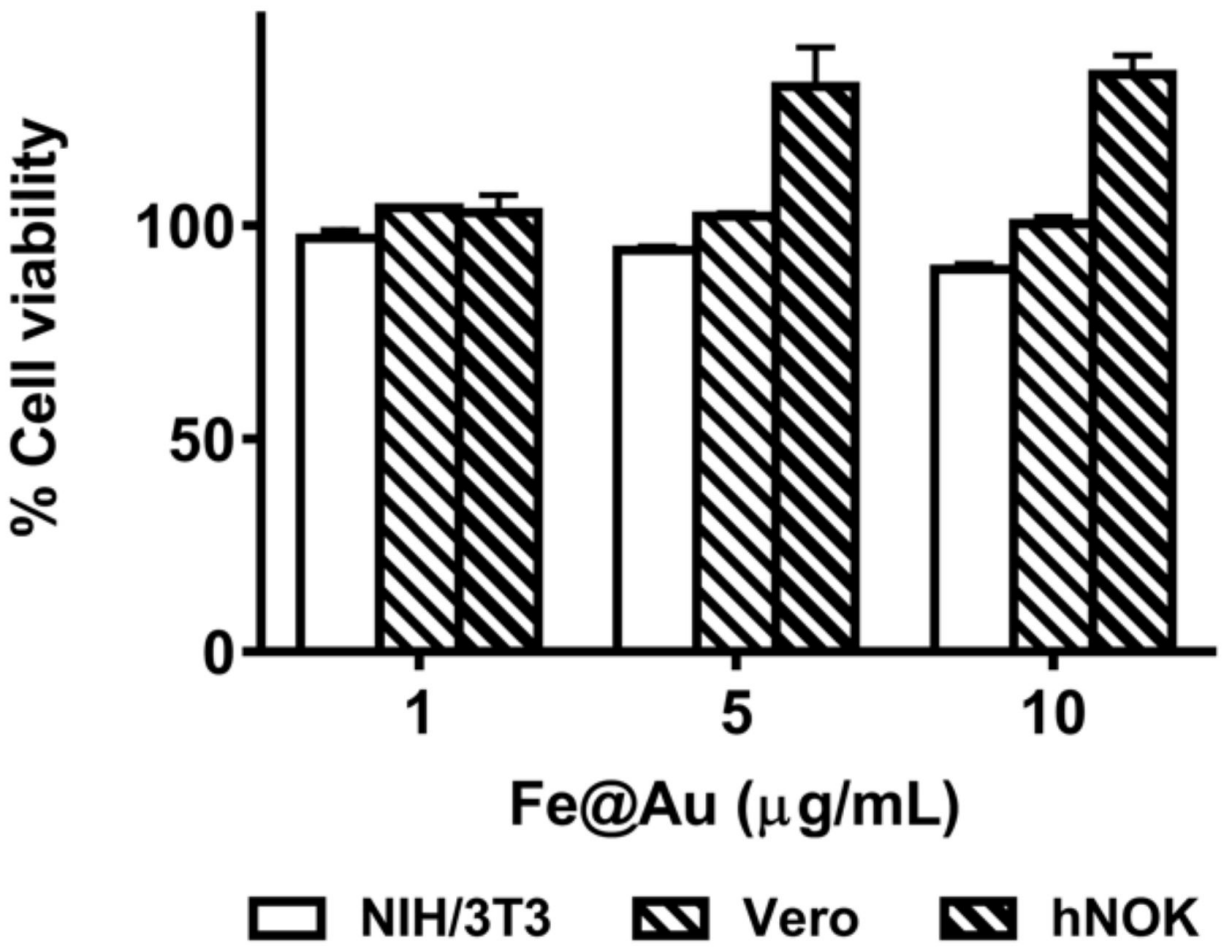
The aged Fe@Au shows great biocompatibility in three different cultured lines of healthy or benign cells. The cell lines, NIH/3T3, Vero and hNOK, were exposed to aged Fe@Au for 48 h and the cell viability was assessed by WST-1 assay. Aged Fe@Au is highly biocompatible in these cells.

To investigate the effect of ageing of Fe@Au nanoparticles on their biocompatibility with healthy cells, we co-cultured different benign cells (NIH/3T3, Vero and hNOK) with aged Fe@Au (Figure 5). There was no significant decrease in cell viability in these three different benign cell lines at doses of up to 10 µg/mL. This is unsurprising in that Fe_2_O_3_and gold is known to be highly biocompatible. However, this supports the hypothesis that Fe@Au nanoparticles degrade into highly biocompatible materials.

### 3.3. Long-Term Storage of Fe@Au Nanoparticles and Preservation of Their Anti-Cancer Properties

**Figure 6:**
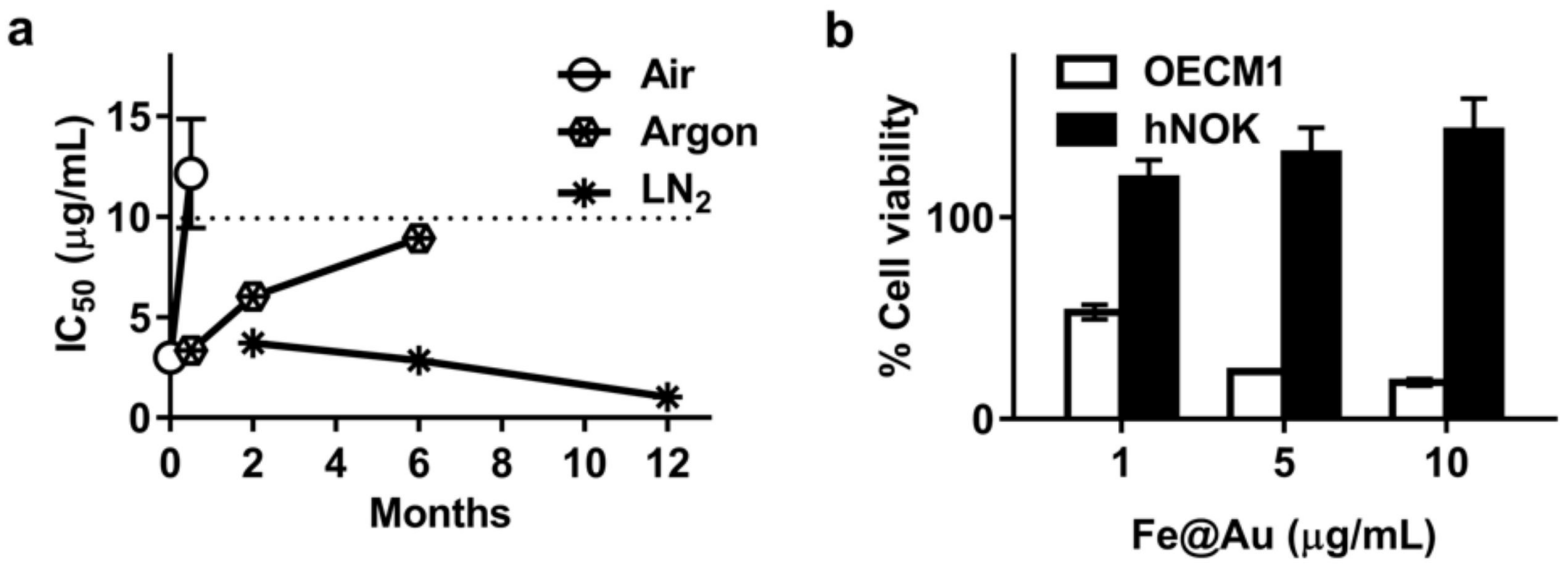
Optimizing the storage condition of Fe@Au to preserve the anti-cancer activity. (**a**) It shows the viability of the oral cancer cell line, OECM1, treated with Fe@Au stored under different conditions—including exposure to air, storage under argon and storage under liquid nitrogen—at different time points. The cell viability was assessed by the WST-1 test after 48 h of treatment. The IC_50_ dosages are calculated according to the cell viability results; (**b**) Fe@Au nanoparticles stored under liquid nitrogen for 12 months were used to treat oral cancer cells, OECM1 and paired healthy cells, hNOK, for 48 h. The cell viability was assessed with the WST-1 assay.

The dramatic decrease in anti-cancer activity of Fe@Au over time compromises their practicality for future clinical applications. We therefore next explored storage under low-oxygen or low-temperature conditions. As shown in Figure 6a, the IC_50_ of Fe@Au exposed to air for two weeks and Fe@Au stored under argon for six months is 12.2 ± 5.4 µg/mL and 8.9 ± 0.5 µg/mL, respectively, while that of freshly reconstituted Fe@Au is 3.0 ± 0.2 µg/mL. These observations illustrate that the anti-cancer effectiveness significantly drops when Fe@Au nanoparticles are exposed to air for two weeks (*p* = 0.0428, Student *t*-test; compared to freshly synthesize and reconstituted Fe@Au) or stored under argon for 6 months (*p* < 0.0001, Student *t*-test; compared to freshly synthesized and reconstituted Fe@Au). Fe@Au nanoparticles stored under argon fare better than those particles exposed to air for two months (*p* = 0.2416, Student *t*-test; compared to freshly synthesize and reconstituted Fe@Au).

Environmental moisture and higher temperatures generally accelerate oxidation, as described by the kinetic Arrhenius equation [30]. Therefore, we trialed storing Fe@Au nanoparticles at low temperatures to reduce oxidation and prolong their efficacy. As shown in Figure 6a, Fe@Au stored under liquid nitrogen for up to 12 months much retains better anti-cancer activity than Fe@Au stored under argon for up to six months, without affecting healthy cell growth (Figure 6b). Indeed, the nanoparticles stored under liquid nitrogen for 12 months showed a gradual increase in cytotoxicity to cancer cells (Figure 6a; *p* < 0.0001, Student *t*-test when compared to Fe@Au stored under liquid nitrogen for only 2 months and *p* = 0.0001, Student *t*-test; compared to freshly reconstituted Fe@Au). They also enhanced the growth of health control cells (hNOK) by 43% at the dosage of 10 µg/mL Fe@Au (Figure 6b; *p* = 0.0068, Student *t*-test; compared to the control group without any Fe@Au treatment).

## 4. Discussion

In our previous work, we have demonstrated that Fe@Au NPs selectively suppress cancer cell growth and left healthy control cells unaffected in vitro and in vivo. The particle treatment caused delay in cell-cycle progression, especially in the S-phase. Besides, Fe@Au causes an irreversible membrane-potential loss in the mitochondria of cancer cells but, at the same time, only a transitory decrease in membrane potential in healthy control cells was observed [19]. Briefly, the ion elements, before oxidation, triggered mitochondria-mediated autophagy and is hence responsible for the differential cytotoxicity observed between cancerous versus healthy cells [21,31]. In this study, we aimed firstly to unravel the underlying mechanisms of the Fe@Au aging process. And secondly, to disclose the correlation between structural/compositional transformation and the anti-cancer properties of the Fe@Au nanoparticles. 

**Scheme 3:**
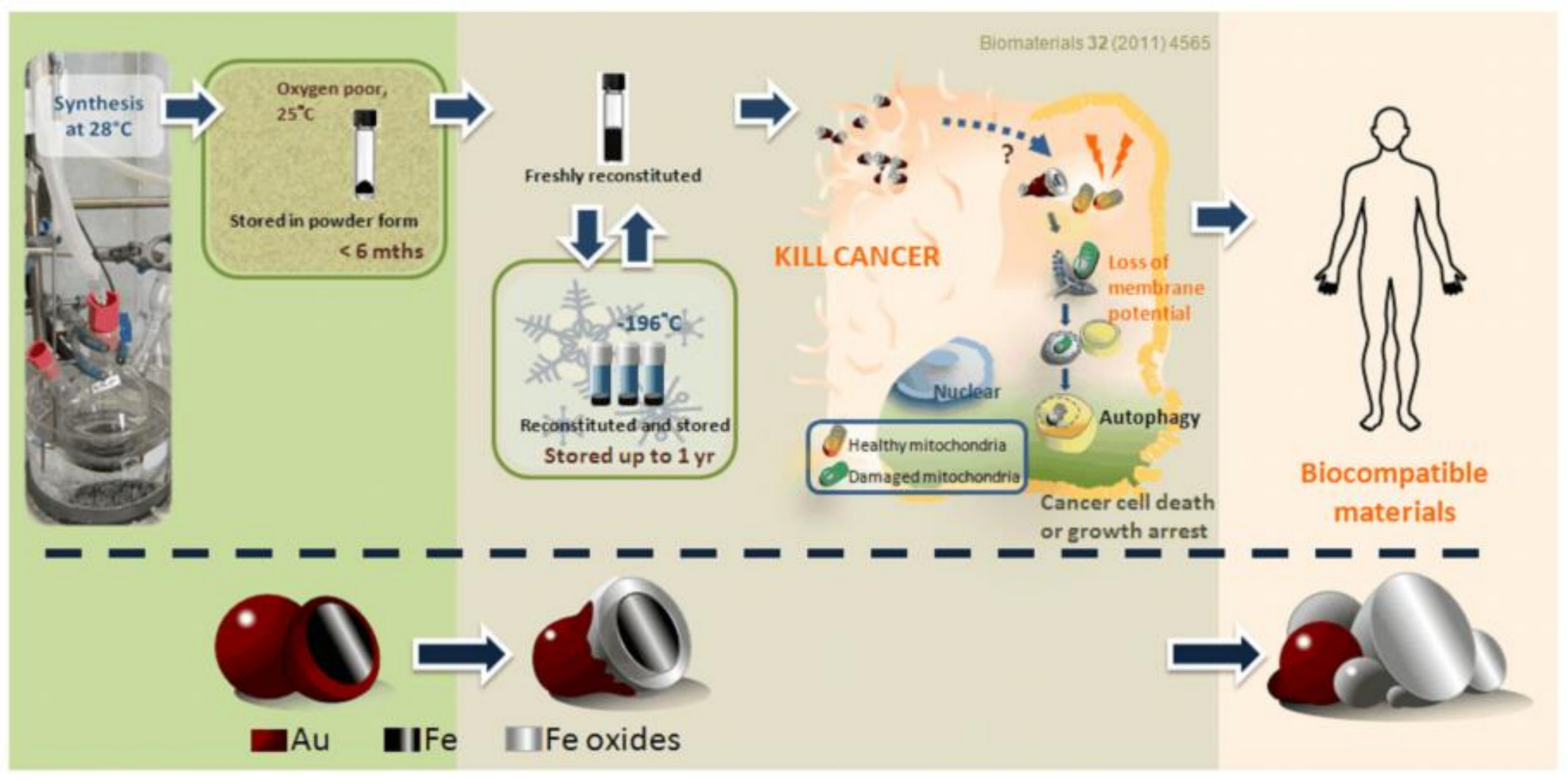
Schematic summarizing the structure of Fe@Au in different stages, with different functionalities, was described.

Despite the high-quality core–shell structure of freshly synthesized Fe@Au, morphological changes of Fe@Au quickly occur after storage and reconstitution for use. The gold coatings tend to agglomerate into small gold nanoparticles and clumps and the Fe cores are exposed, as we observed with electron microscopy (Figure 1b). The ring-like pale layers found around the electron dense cores (Figure 1b, black arrows) are similar to electron microscopy data reported by others who have found that the oxidation of Fe nanoparticles led to either vacancies or Fe-cores covered by layers of iron oxide in a low-contrast laminated structure [32]. In terms of the cytotoxic activity of the nanoparticles, it seems probable that the exposure of the Fe cores is at least partially initiated during the reconstitution step and that this helps activate the subsequent cytotoxic effects that are caused by the reducing properties of the Fe(0). During the uptake and internalization of the nanoparticles by the cancer cells, the Fe cores are oxidized, creating the void structures we observed within the cancer cells, as is apparent in Figure 2; indeed these void structures were evident in the cluster of Fe@Au within lysosomes after 24 h’ treatment. Such voids are consistent with the work of Cabot et al. in which Fe nanoparticles formed hollow particles during oxidation as the Fe atoms rapidly diffused to the particle surfaces to become part of the growing oxide layer [33]. Cabot et al. and Wang et al. reported that Fe nanoparticles with a size of approximately 8 nm or below would become fully oxidized and form a completely hollow center, whereas larger particles retained elemental Fe within the oxide coating [32,33]. The particle growth indicated from our XRD data of aged and fresh Fe@Au nanoparticles is also consistent with the diffusion of Fe to the particle surfaces during oxidation. Thus, it appears that the protective Au shell is partially damaged by reconstitution, which exposes the Fe cores and initiates the oxidation process and probably causes further exfoliation of the remaining Au from the nanoparticles as the exposed Fe continues to oxidize within cancer cells (Scheme 3). 

Another interesting observation from our detailed storage assessment studies is that healthy oral cells (hNOK cell line) showed increased cell proliferation when treated with Fe@Au nanoparticles (Figure 5 and Figure 6A). Indeed, it has been reported that metal oxides, that is, ZnO [34], Ag/ZnO [35] and TiO_2_ [36], enhanced wound healing in biological systems. Augustine et al. proposed that the pro-inflammation properties of metal oxides triggered the proliferation of fibroblasts and thus promote wound healing [37]. To date there is still limited literature available investigating nanoparticle-mediated cell growth and it is unclear at present what mechanism is responsible. Nonetheless, this is a phenomenon that deserves attention in the near future, especially if such core–shell nanoparticles are developed for use as cancer treatments.

The other key point to note is that, from our XRD data (Figure 4), ageing of Fe@Au produces γ-Fe_2_O_3_, which is highly biocompatible, as confirmed by our cytotoxicity test of aged Fe@Au on three benign or healthy cell lines (Figure 5). Conversion of the Fe(0) to hematite also accounts for why aged Fe@Au nanoparticles lose their cytotoxicity toward cancer cells, as we have shown in our previous reports [19,21]. This ageing means that careful storage is necessary (in low-oxygen conditions and ideally frozen at liquid-nitrogen temperatures) to maintain the efficacy of the Fe@Au nanoparticles. Still it is important to recognize that the spontaneous decomposition of Fe@Au nanoparticles into iron oxides and gold after anti-cancer treatments is a boon for potential future clinical use as it means that the nanoparticles should inactivate themselves during their therapeutic action, by the oxidation of the Fe(0). We consider that this could offer an attractive alternative to, or adjunct with, current chemotherapies, which often have highly undesirable side effects. This in-built inactivation or “self-detoxification” in use, potentially without applying any load to patients’ kidneys or livers, offers a different concept in drug design. 

## 5. Conclusions

Clearly the structural transformation of Fe@Au upon oxidation during prolonged storage is correlated with their decreasing anti-cancer activity. Our findings confirm other work showing that the gold coatings of Fe@Au serve to protect the iron cores from rapid oxidation in the short-term [16] but extended storage requires greater care to retain their anti-cancer activity. Optimal storage seems to be frozen in ethanol suspension in liquid nitrogen. Indeed, we showed that the anti-cancer activity of Fe@Au can be preserved for at least one year when stored under liquid nitrogen and that the cytotoxicity for cancer cells actually increases slightly over this time, while the biocompatibility for healthy cells is retained. From the perspective of possible future clinical usage, it is beneficial that, upon ageing in air or when introduced into cells, the Fe@Au nanoparticles decompose into irregular fragments of γ-F_2_O_3_ and Au, both of which are considered highly biocompatible and are approved for in-human use by the FDA.

For the future, the practical use of Fe@Au as a low-toxicity anti-cancer drug will require further investigation. For example, the next stages should be further detailed preclinical (animal model) studies to assess the interaction between Fe(0)-containing nanoparticles and the major detoxifying organs and then clinical trials of the nanoparticles. Despite the work still to be done, the high efficacy of Fe@Au against cancer cells and inherent high biocompatibility indicate the strong potential that the particles hold as a Fe(0)-based novel anti-cancer therapy.

## Figures and Tables

**Table 1:** Elemental compositions determined from EDXS (see also, Supplementary Figure S1).

Element	Weight (%) ± Standard Dev.	Atomic (%)
(a) Freshly prepared Fe@Au, as shown in Figure 1a		
Fe K	20.6 ± 0.4	47.9
Au L	79.4 ± 0.4	52.1
(b) The dense area of reconstituted Fe@Au, as marked with white asterisk in Figure 1b		
Fe K	3.7 ± 0.3	12.0
Au L	96.3 ± 0.3	88.0
(c) A less-dense area of reconstituted Fe@Au, as marked with black asterisk in Figure 1b		
Fe K	61.2 ± 1.7	37.0
Au L	13.8 ± 1.6	1.7

## Supplementary Materials

The following are available online at http://www.mdpi.com/1996-1944/11/12/2572/s1, Figure S1: The quality control of the cancer selective anti-cancer effect of Fe@Au, Figure S2: Investigation of synthesized Fe-core-alone particles by synchrotron radiation based XRD, Table S1: The Fe/O atomic ratio of reconstituted and aged Fe@Au, EDXS.
