# Supplementary material for: Characterization of Iron Core–Gold Shell Nanoparticles for Anti-Cancer Treatments: Chemical and Structural Transformations During Storage and Use
Source: Materials (Basel). 2018 Dec 17;11(12):2572. doi: 10.3390/ma11122572 (PMC6316008; doi:10.3390/ma11122572)
Supplement: Supplementary file 1 [file materials-11-02572-s001.pdf]

materials

Supplementary information

Characterisation of Iron Core–Gold Shell Nanoparticles for Anti-Cancer Treatments: Chemical and Structural Transformations During Storage and Use

Ya-Na Wu[1,2,*,†], Dar-Bin Shieh[1, 3], Li-Xing Yang[4], Hwo-Shuenn Sheu[5], Rongkun Zheng[6], Pall Thordarson[7], Dong-Hwang Chen[8], and Filip Braet[6, 9]

1 Institute of Oral Medicine and Department of Stomatology, College of Medicine, National Cheng Kung University Hospital, National Cheng Kung University, Tainan, 70101, Taiwan; dbshieh@mail.ncku.edu.tw
2 Institute ofBiological Chemistry, Academia Sinica, Taipei, 11529, Taiwan
3 Center for Micro/Nano Science and Technology, Advanced Optoelectronic Technology Center, Innovation Center for Advanced Medical Device Technology, National Cheng Kung University, Tainan, 70101, Taiwan
4 Institute of Basic Medical Sciences, National Cheng Kung University, Tainan, 70101, Taiwan; fingerzoo@gmail.com
5 National Synchrotron Radiation Research Center, Hsinchu Science-Based Industrial Park, Hsinchu 30076, Taiwan;hsheu@nsrrc.org.tw
6 Australian Centre for Microscopy & Microanalysis, The University of Sydney, Sydney, NSW 2006, Australia; rongkun.zheng@sydney.edu.au
7 School of Chemistry, The University of New South Wales, Sydney, NSW 2052, Australia; p.thordarson@unsw.edu.au
8 Department of Chemical Engineering, National Cheng Kung University, Tainan 70101, Taiwan; chendh@mail.ncku.edu.tw
9 School of Medical Sciences (Discipline of Anatomy and Histology)—The Bosch Institute, The University of Sydney, NSW 2006, Australia; filip.braet@sydney.edu.au
* Correspondence: yana.wu@gmail.com; Tel.: +886-6-235-3535 ext. 5377

Received: date; Accepted: date; Published: date

Materials and Methods:

Characterization of freshly synthesized Fe@Au

Fe@Au nanoparticles were diluted into ethanol and quickly applied onto copper grids coatedwith a thin carbon support film. The specimens were immediately observed at an accelerating voltageof 300 kV using a 3000F TEM (JEOL, Tokyo, Japan), equipped with a SC1000 11-megapixel CCD camera (Gatan, CA, USA). X-ray diffraction (XRD) analysis of the nanoparticles was done using Cu/K α-radiation, generated at 40 kV and 30 mA (average wavelength is 0.15418 nm), in a Bruker X-ray diffractometer (RX-III; Shimadzu Corp., Kyoto, Japan). XRD patterns ($20^{\circ} < 2\theta < 90^{\circ}$) were collected at a scan rate of 5° per minute.

Fe/O atomic ratio of reconstituted and aged Fe@Au

Fe@Au nanoparticles were diluted into ethanol and quickly applied onto copper grids coated with a thin carbon support film. The specimens were immediately observed at an accelerating voltage of 200 kV using a 2100F TEM (JEOL, Tokyo, Japan), equipped with anEDXS system. The mean Fe/O ratio of reconstituted and aged Fe@Au is 0.447 ± 0.07 and 0.314 ± 0.09, respectively, which is significantly different ($p = 0.0001$, Student's T test).

Results:

Table S1. The Fe/O atomic ratio data of reconstituted and aged Fe@Au obtained through EDXS. Each line of the table is the data from a single-field EDXS analysis of the two samples, with the mean ± standard error given in the final row in bold.

Reconstituted Fe@Au, atomic %			Aged Fe@Au, atomic %		
O	Fe	Au	O	Fe	Au
64.5	23.5	11.1	63	25.5	11.5
41.1	20.8	38.1	46.4	20.7	33
61.5	26.1	12.4	62.7	20.9	16.4
51.4	26.4	22.2	61.7	21.6	16.7
61.4	23.3	15.2	63.1	17.5	19.4
55.1	22.2	22.8	68.2	20.6	11.2
53.4	30.1	16.4	60.6	23.7	15.8
57.9	25.1	19	48.2	19	32.8
50	24.4	25.6	61	25.6	13.4
40.3	18.7	40.9	60.7	5.5	33.7
65.4	23.5	11.1	65.6	19.6	14.8
45.6	23.8	30.6	69.9	17.8	12.3
63.3	24.4	12.4	66.8	14.7	18.5
			70.6	17.5	11.9
			57.4	23.6	19
			61.7	19.8	18.5
			62	23.6	14.4
			60.8	17.4	21.8
			60.9	18.8	20.3
			75.7	10.3	14
54.7 ± 2.4	24.0 ± 0.8	21.4 ± 2.8	61.4 ± 1.9	19.4 ± 1.4	19.2 ± 2.3

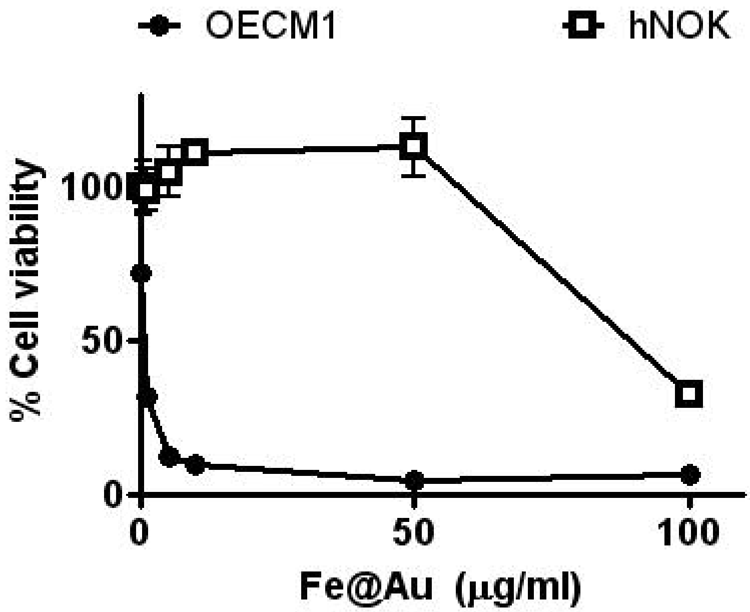

Figure S1. Quality control data on the anti-cancer selective effects of Fe@Au. Prior to further experiments, the cancer specific anti-cancer effect of Fe@Au was estimated by WST-1 assay with 48 hours' treatment. The IC_{50} of the freshly synthesized Fe@Au in OECM1 is between 1 and 5 μg/mL, while a significant decrease in cellular viability was only observed at doses exceeding 50 μg/mL in hNOK cells ($p = 0.0053$, Student t-test; compared to Fe@Au-treated OECM1).

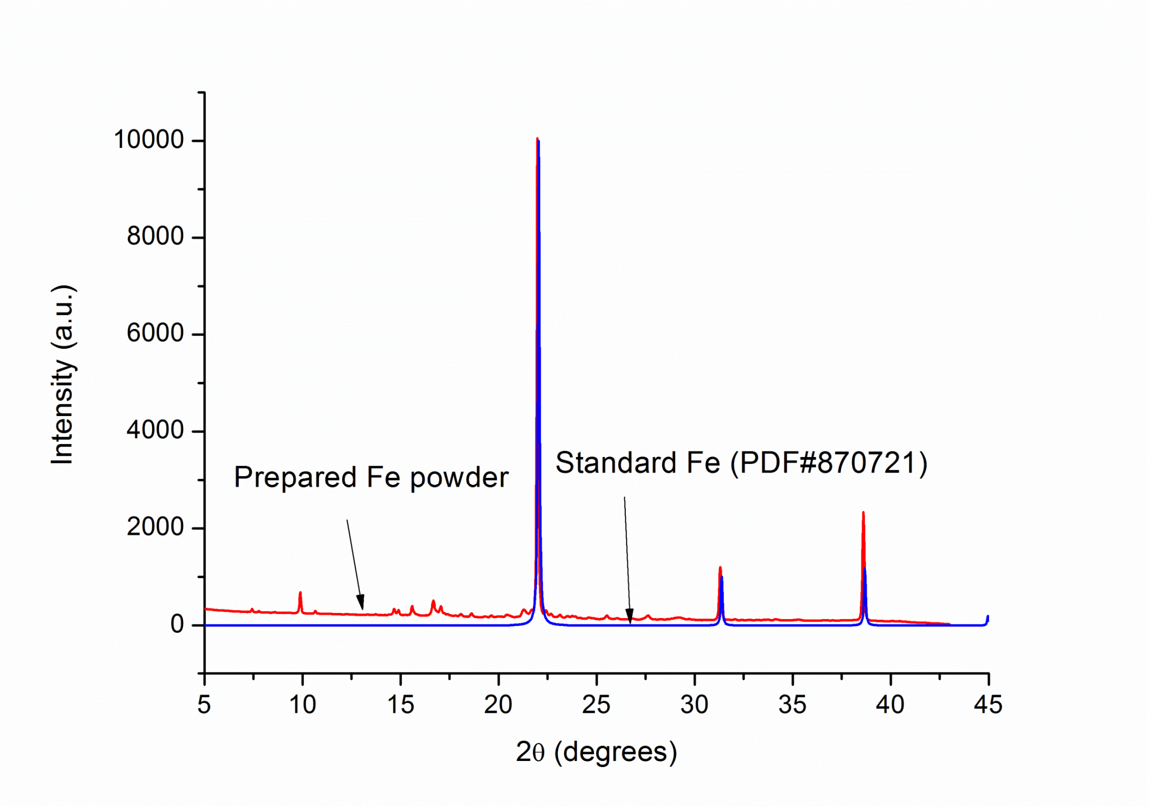

Figure S2. Investigation of synthesized Fe-core-alone particles by synchrotron radiation based XRD. To avoid the problems of overlapping peaks from Fe and Au under XRD of Fe@Au, we synthesized pure Fe core nanoparticles and analyzed them by XRD. This shows that the major peaks are identical with the standard powder diffraction file of elemental Fe.
